# Cryptococcal antigen screening by lay cadres using a rapid test at the point of care: A feasibility study in rural Lesotho

**DOI:** 10.1371/journal.pone.0183656

**Published:** 2017-09-06

**Authors:** Fernanda Rick, Aline Aurore Niyibizi, Amir Shroufi, Kazumi Onami, Sarah-Jane Steele, Malehlohonolo Kuleile, Innocent Muleya, Tom Chiller, Tiffany Walker, Gilles Van Cutsem

**Affiliations:** 1 Médecins Sans Frontières, Cape Town, South Africa; 2 Médecins Sans Frontières, Maseru, Lesotho; 3 Mycotic Diseases Branch, Centers for Disease Control and Prevention, Atlanta, Georgia, United States of America; 4 Epidemic Intelligence Service, Centers for Disease Control and Prevention (CDC), Atlanta, GA, United States of America; 5 Centre for Infectious Disease Epidemiology and Research, University of Cape Town, Cape Town, South Africa; Aga Khan University Hospital Nairobi, KENYA

## Abstract

**Introduction:**

Cryptococcal meningitis is one of the leading causes of death among people with HIV in Africa, primarily due to delayed presentation, poor availability and high cost of treatment. Routine cryptococcal antigen (CrAg) screening of patients with a CD4 count less than 100 cells/mm^3^, followed by pre-emptive therapy if positive, might reduce mortality in high prevalence settings. Using the cryptococcal antigen (CrAg) lateral flow assay (LFA), screening is possible at the point of care (POC). However, critical shortages of health staff may limit adoption. This study investigates the feasibility of lay counsellors conducting CrAg LFA screening in rural primary care clinics in Lesotho.

**Methods:**

From May 2014 to June 2015, individuals who tested positive for HIV were tested for CD4 count and those with CD4 <100 cells/mm^3^ were screened with CrAg LFA. All tests were performed by lay counsellors. CrAg-positive asymptomatic patients received fluconazole, while symptomatic patients were referred to hospital. Lay counsellors were trained and supervised by a laboratory technician and counsellor activity supervisor. Additionally, nurses and doctors were trained on CrAg screening and appropriate treatment.

**Results:**

During the study period, 1,388 people were newly diagnosed with HIV, of whom 129 (9%) presented with a CD4 count <100 cells/mm^3^. Of these, 128 (99%) were screened with CrAg LFA and 14/128 (11%) tested positive. Twelve of the 14 (86%) were asymptomatic, and received outpatient fluconazole. All commenced ART with a median time to initiation of 15.5 days [IQR: 14–22]. Of the asymptomatic patients, nine (75%) remained asymptomatic after a median time of 5 months [IQR; 3–6] of follow up. One (8%) became co-infected with tuberculosis and died and two were transferred out. The two patients with symptomatic cryptococcal meningitis (CM) were referred to hospital, where they later died.

**Conclusions:**

CrAg LFA screening by lay counsellors followed by pre-emptive fluconazole treatment for asymptomatic cases, or referral to hospital for symptomatic cases, proved feasible. However, regular follow-up to ensure proper management of cryptococcal disease was needed. These early results support the wider use of CrAg LFA screening in remote primary care settings where upper cadres of healthcare staff may be in short supply.

## Introduction

Approximately a third of patients initiating antiretroviral therapy (ART) in sub-Saharan Africa present with advanced HIV, defined WHO disease stage 4 and/or a CD4 count <100 cells/mm^3^ [1]. This group is at particular risk for opportunistic infections (OI). Of these OIs, cryptococcal meningitis (CM) and tuberculosis (TB) are of particular importance, accounting for the majority of HIV-related deaths [2,3]. CM is estimated to cause 350,000 deaths per year, with case fatality rates ranging from 35–65% in sub-Saharan African settings [1]. Even in developed countries, CM is still considered a severe OI, with an estimated case fatality rate of 10–20% [2,4].

Many deaths from cryptococcal disease are attributed to delays in seeking healthcare, resulting in diagnosis when disease has already progressed to an advanced stage, at which point available treatments are less effective. Limited access to rapid diagnostic tests and materials for lumbar punctures (LPs) add to these delays [5]. Access to treatment is often challenging following diagnosis, due largely to the high cost and limited availability of intravenous amphotericin B, the recommended induction treatment for CM. Moreover, patients taking amphotericin B must be closely monitored for toxicity and complications, which can be challenging in resource-limited settings.

Early initiation of ART in CM patients can result in potentially life-threatening immune reconstitution syndrome (IRIS), and for that reason it is recommended that ART is delayed by 4–6 weeks following effective antifungal therapy. However, the presence of CrAg in the blood is highly predictive of future development of CM [6–8], and many CM and IRIS cases that manifest after ART initiation could be prevented by screening and treating asymptomatic CrAg-positive individuals with fluconazole as outpatients [8–11]. This aligns with the concept of the ‘test-and-treat’ strategy.

In countries where CD4 testing is predominantly laboratory based, several strategies to increase CrAg screening among at-risk patients are possible, such as laboratory-based “reflex” testing. This strategy entails automatic testing for CrAg among patients with a CD4 count <100 cells/mm3, using remnant serum from the CD4 tests. South Africa is currently doing this, in line with the National Strategic Plan for HIV, STIs and TB 2012–2016 [12].

Point-of-care (POC) testing using CrAg LFA offers an alternative strategy, allowing for immediate intervention. The CrAg LFA test has demonstrated high sensitivity (100%) and specificity (ranging from 94–100%) when used with whole blood, serum or cerebrospinal fluid (CSF) [13–15]. Additionally, test kits are relatively inexpensive (approximately US$2), and tests can be done with minimally invasive sampling (i.e. finger-prick whole blood, plasma/serum) [8,15]. The CrAg LFA test also meets the World Health Organization (WHO) ASSURED criteria for diagnostics for resource-limited countries, being **A**ffordable, **S**ensitive, **S**pecific, **U**ser-friendly, **R**apid, **E**quipment-free and **D**eliverable to those who need it [5]. This ability of a diagnostic test to perform at the point of care offers particular promise for diagnosis of cryptococcal disease in rural, resource-limited settings. POC testing would allow medical staff to begin same-day treatment, avoiding delays in treatment initiation and curbing loss to follow-up. Moreover, instrument-free tests such as LFAs allow for testing at sites with little or no infrastructure, electricity or refrigeration. Despite the ease of use of this test, few data exists on the potential role of systematic CrAg LFA screening at the point of care in ART clinics in sub-Saharan Africa. In order to address this knowledge gap, CrAg LFA screening at the point of care was piloted and evaluated in Lesotho.

Lesotho, with a population of 2.1 million, is a small highland country with the third highest HIV prevalence in the world, at 23.2% among adults aged 15–49 [16]. ART was introduced in the public sector in 2004. In 2015, the estimated number of people living with HIV was 310,000 and the estimated number of AIDS-related deaths was 9,900 [17].

Lesotho’s population is largely rural, with 70% of people living outside of cities [18]. Laboratory-based CD4 measurements are available at two of the country’s district hospitals, and a limited number of primary care clinics have access to point-of-care CD4 technology such as the PIMA POC CD4 analyser (Alere Inc., Waltham, MA, USA). Primary health care clinics in Lesotho are staffed mainly by nurses and lay counsellors, without regular doctor assistance. Additionally, referral from the clinic to the district hospital level is challenging due to the mountainous nature of the country and the lack of reliable ambulance or public transportation in rural areas.

The prevalence of CrAg in patients entering ART programs in Lesotho has not been reported to date. Screening has not been implemented despite recommendation by the WHO and the existence of national guidelines that provide for this, which means that cases often go unidentified and untreated. Slow adoption may be attributable to human resource constraints, in particular the severe shortage of qualified healthcare workers.

In the Roma- Semonkong area, most patients on ART are managed at the clinic level and in some clinics CD4 testing for ascertaining ART eligibility is done at the point of care by lay counsellors using PIMA machines. Given the widespread use of PIMA in Roma-Semonkong sub-district and the familiarity of lay counsellors with POC technology, the district is an ideal setting for the introduction of POC CrAg LFA screening.

### MSF in Lesotho

Since May 2014, MSF has supported routine screening using CrAg LFA followed by pre-emptive treatment of positive patients with outpatient fluconazole in 3 rural health facilities in the Roma-Semongkong sub district of Maseru district, Lesotho. Among our catchment population all clinics had access to PIMA, whereas nationwide PIMA access is variable and difficult to quantify due to the absence of national level data. This study describes the feasibility of implementing a screen-and-treat strategy for cryptococcal disease using lay counsellors and nurses in rural primary care clinics.

## Methods

Screening of all HIV-infected patients with a CD4 count <100 cells/mm^3^ with the POC CrAg LFA test (Immy, Inc., Norman, OK, USA) was implemented in three primary care rural clinics in the Roma- Semonkong sub-district (St. Leonard, Nazareth and Fatima), where the PIMA POC CD4 analyser was readily available.

In order to reduce nurse workload and improve patient flow, lay counsellors were trained to perform CrAg-LFA testing on capillary whole blood.

A laboratory technologist trained several lay counsellors to collect finger-prick specimens, to read and record CrAg LFA results and to dispose of materials in a safe, bio-hazardous waste bin. Training materials included visual support aids, package inserts and standard operating procedures (SOPs) developed jointly with the Ministry of Health (MoH) Quality Assurance (QA) Office.

**Fig 1:**
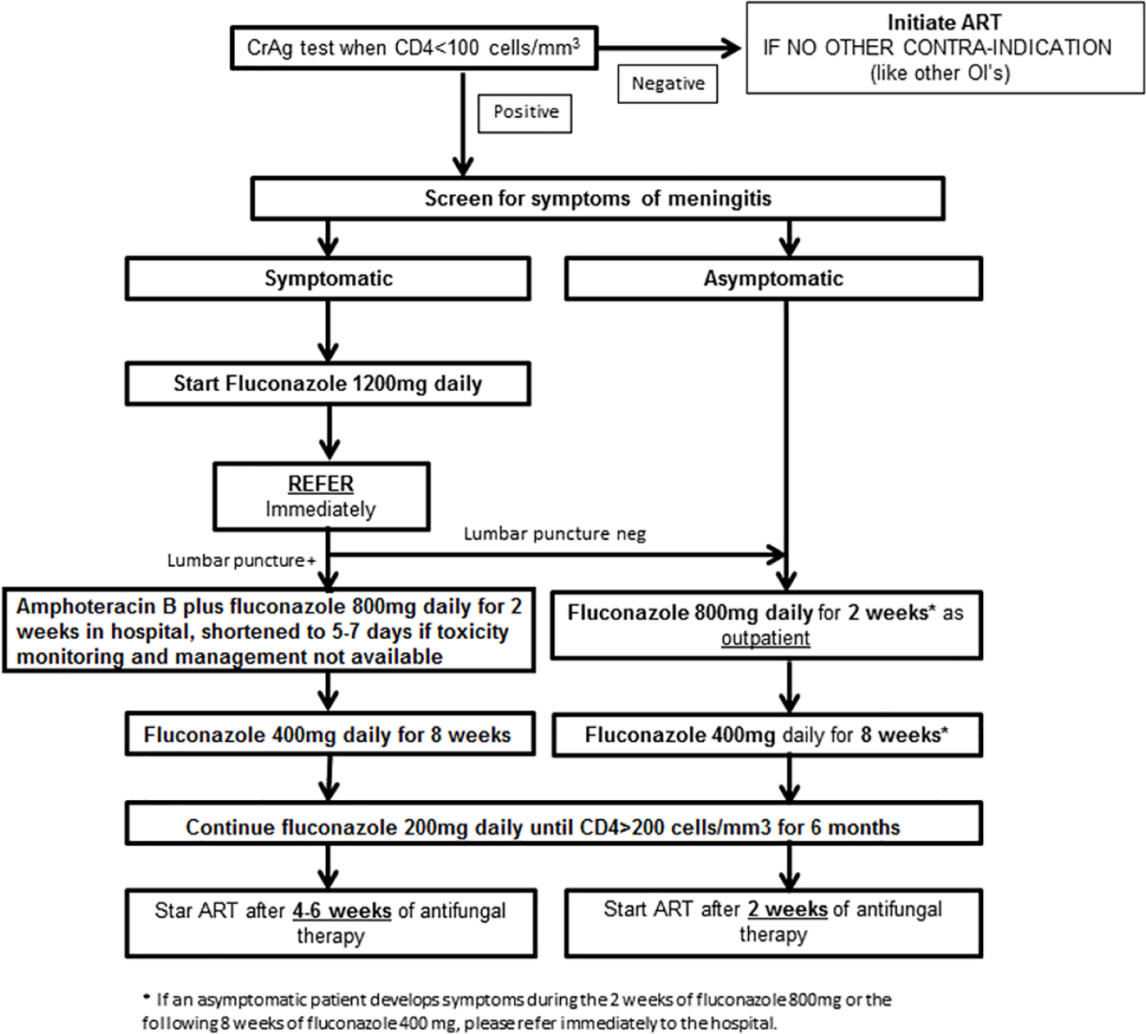
Algorithm for screening and pre-emptive treatment of cryptococcal meningitis.

A CrAg register was provided to each clinic for result recording. As part of on-going monitoring and supervision, a laboratory technologist and a counselor supervisor visited the clinics monthly to ensure adherence to SOPs and good clinical practice. To ensure that CrAg-positive patients received proper clinical management at the time of presentation, nurses were trained to appropriately treat asymptomatic disease and to refer symptomatic patients. Likewise, doctors at the referral hospital were provided with training in diagnosing and treating CM (Fig 1).

Lay counsellors collected finger-prick specimens and performed CrAg-LFA testing in designated HIV counseling and testing (HCT) rooms. CrAg LFA testing was performed according to the manufacturer’s instructions.

In order to ensure appropriate performance of all test strips included in each box, CrAg-positive and negative controls were performed for each box of tests. If both positive and negative control samples were correctly identified by the LFA, then all test strips included in that box were deemed to be working and ready for use.

In line with WHO’s Rapid Advice on diagnosis, prevention and management of cryptococcal disease in HIV-infected adults, adolescents and children, CrAg screening was carried out for ART-naïve HIV positive adults (>18 years old) with a CD4 count <100 cell/mm^3^ [5,19]. CrAg-negative patients were initiated on ART as soon as possible by nurses trained in HIV management (OIs), in accordance with national guidelines [16].

Nurses were trained on the signs and symptoms of CM, including fever, headache, altered mental status, seizure, and neck stiffness [16]. CrAg-positive individuals were investigated for these signs or symptoms. In the presence of any sign or symptom of CM, patients were referred to the district hospital for clinical assessment, LP and CSF testing using the CrAg LFA. In confirmed CM cases (CrAg LFA positive on CSF), patients were started on intravenous amphotericin B deoxycholate 1mg/kg/day for 2 weeks initially. Given that access to renal function monitoring was not available, we later recommended that a short course of amphotericin B deoxycholate 1mg/kg for 5–7 days be used in order to reduce the risk of renal failure [5]. In additional to amphotericin, oral fluconazole 800mg was given once daily while the patient was in hospital, followed by 400mg daily for 8 weeks as an outpatient. Patients without signs or symptoms of CM or with CrAg-LFA-negative results on CSF were started on fluconazole 800mg once daily for 2 weeks, followed by fluconazole 400 mg once daily for 8 weeks. In all cases, fluconazole 200 mg maintenance phase was given daily until CD4>200, and for at least 6 months. All results for CrAg-LFA tests done from May 2014 to June 2015 were recorded, and these dates constitute the study period. The main outcomes were defined as “alive and in care”, “dead”, “transferred out” (to another health facility) or “lost-to-follow-up” (LTFU). The CrAg registers at participating sites were reviewed on a weekly basis and all anonymised patient data was entered into an Epi-Info database. Statistics were performed using STATA version 13 (StataCorp, Texas, USA).

This pilot project was carried out after screening for cryptococcal disease had been incorporated into Lesotho national guidelines. We applied for ethical approval for this work to the Lesotho National Heath research ethics committee. The committee exempted the proposal from research ethics review on the basis that it constituted the implementation of national guidelines. Because we were implementing something that was already part of national guidelines, participants were not asked to provide informed consent to participate, and this was not asked for by the ERB. The only patient samples used were finger-prick blood specimens for routine care, collected by Lesotho lay counsellors for CrAg LFA testing. No samples were stored or collected for any other reason.

## Results

Demographic and clinical data for the 128 CrAg-LFA-screened people are presented in Table 1.

**Table 1:** Main characteristics of patients according to CrAg- LFA results.

	CrAg-LFA negative	CrAg-LFA positive	p
**No. patients, N (%)**	114 (89)	14 (10.9)	-
**Mean Age (**±**SD)**	36 (12)	39 (13)	0.442
**Mean CD4 count (cells/mm**^**3**^**)**	51	50	0.89
**Male, N (%)**	61 (53.5)	13 (92.8)	**0.005**

The median age was 35, (SD 12; range 18–64). There was no difference in the mean age and mean CD4 count between patients testing positive or negative for CrAg. However, there was a statistically significant association with gender, with all but one of those testing positive being male (13/14). Median CD4 count at presentation for males was 50 cells/mm^3^ compared with 53 cells/mm^3^ in females (p = 0.6).

**Fig 2:**
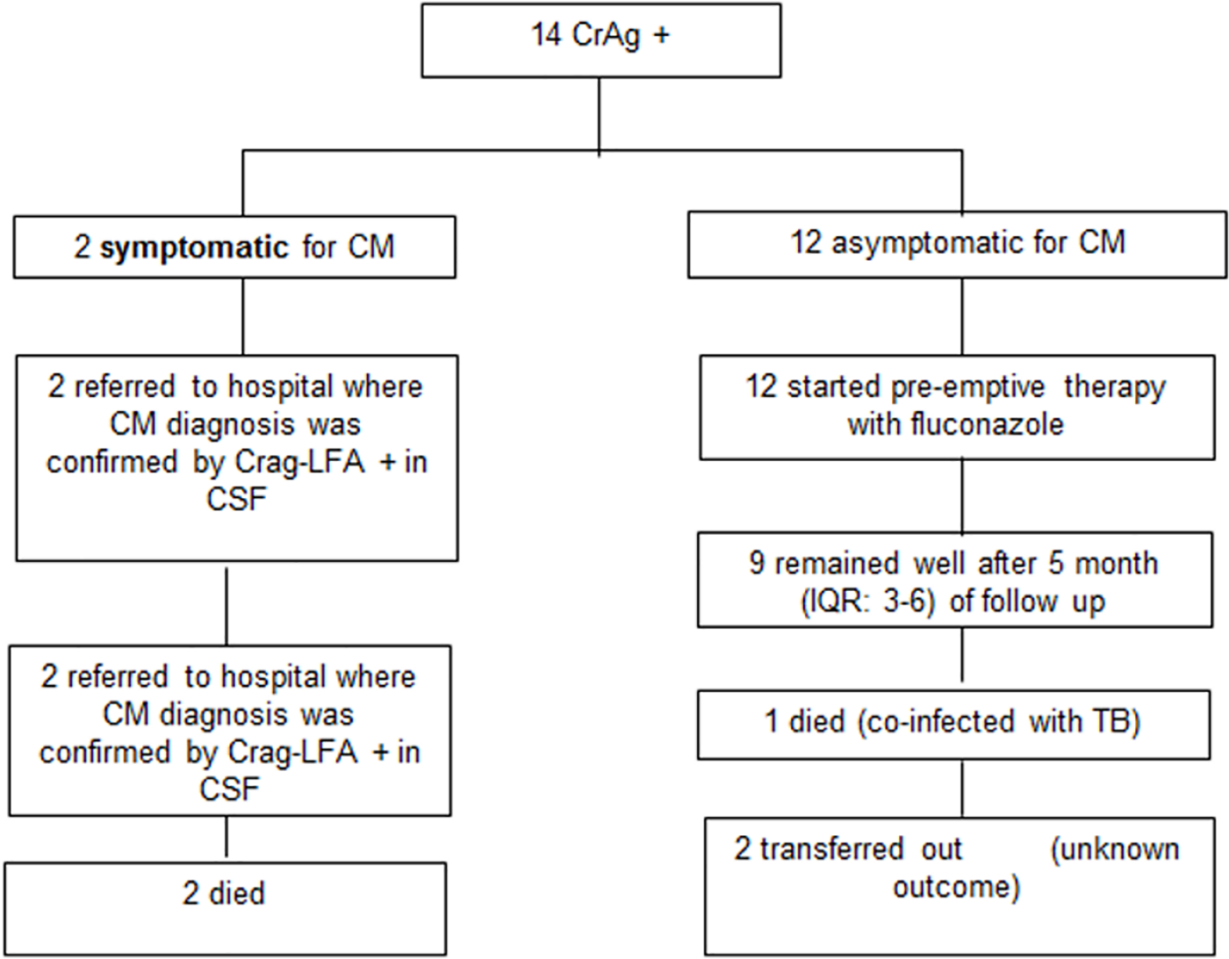
Outcomes among patients who tested positive for CrAg-LFA.

During the study period, 1,388 people were newly diagnosed with HIV in the implementation sites, of whom 129 (9%) had a CD4 count <100 cells/mm^3^. Of these, 128 (99%) were screened with CrAg-LFA, and 14 (11%) tested positive (Fig 2).

Twelve CrAg-LFA-positive persons were asymptomatic, and received outpatient fluconazole (Fig 1). All were subsequently started on ART after a median of 15.5 days [IQR: 14–22]. Nine (75%) remained well after a median of 5 months [IQR: 3–6] of follow up; one (8%) became co-infected with tuberculosis and died, and two (17%) were transferred out.

The remaining two CrAg-positive patients were diagnosed with CM and were referred to hospital where they subsequently died.

One patient died during the follow up period, among those that tested CrAG positive but without showing symptoms of meningitis.

## Discussion

We describe the successful implementation of point-of-care testing by lay counsellors and pre-emptive treatment of asymptomatic CrAg positive patients by nurses at primary care clinics in rural Lesotho. CrAg was detectable in 11% of patients with CD4 counts <100 cells/mm^3^. This is the first CrAg prevalence data from ART clinic attendees in Lesotho, and although considerably higher than the prevalence observed in neighboring South Africa [6], these findings are broadly consistent with those from the wider region, ranging from 3 to 16% [7,9,20–22].

Given the high CrAg prevalence observed and the high likelihood of mortality without pre-symptomatic or early disease identification and treatment, we consider routine screening and targeted pre-emptive antifungal treatment for those testing CrAg-positive to be a worthwhile intervention, particularly in settings like Lesotho, where the prognosis is poor once meningitis develops.

The cost-effectiveness of this intervention has been supported through recent sub-Saharan and Southeast Asian data, which should provide an impetus for policy makers to consider this intervention, even in resource-limited settings [9,23]. Previous work in Uganda showing this strategy to be cost-effective demonstrated that the number that needed to test and treat (NNT) in order to achieve desired cost-effectiveness was 11.3 (95%CI: 7.9–17.1). The NNT to save one life in this study was 15.9 (95%CI: 11.1–24.0), and the cost per disability-adjusted life year (DALY) saved was $21 (95% CI: $15-$32) [9]. Another study in South Africa demonstrated that screen and treat strategy is cost-effective even in areas where CrAg prevalence is as low as 0.6% [24].

To our knowledge, this is the first study showing how CrAg prevalence differs by sex [6,7,9,20]. We observed male predominance of CrAg-positive patients—however, as we had a small sample size this may be a chance finding. Interestingly, it is well established that paracoccidioidomycosis affects men more frequently, which has been attributed to the protective effects of oestrogen [23]. Further studies would be needed to investigate this finding further.

### Implementation challenges

The need for close supervision provided a particular challenge, especially during the initial phase of implementation. Fluconazole treatment involves 3 phases that are conducted over a fairly lengthy amount of time, and adherence to dosing protocols required constant reinforcement. This was complicated by the fact that we were working in remote areas, where access to secondary level care is limited. Furthermore, the relatively low number of CrAg-positive patients restricted the opportunities to supervise and discuss the cases with lay counsellors and clinicians during the consultation time. Achieving a high training coverage of staff was limited by high turnover and workload. Consequently, frequent refresher trainings were needed.

We did not perform LPs on all asymptomatic patients because they were not available in nurse-operated, rural clinics. Hospital referral was difficult, and even at the hospital level it is difficult to ensure that a LP is carried out. Of our 2 referred patients, we have evidence of only 1 receiving a LP. Both, however, received treatment with amphotericin B, and fluconazole at the correct dosages. While more research is needed to investigate optimal indications for LPs, offering systematic LPs to all patients with a positive CrAg would not be feasible at the primary care level in resource-limited settings.

Other challenges included poor documentation by clinicians, which compromised any potential retrospective review of cases. This was a problem particularly in respect of the recording of fluconazole treatment (dose, duration and adherence), outcomes (improvement, death, co-morbidities) and referrals.

The lack of adequate monitoring could contribute to the deaths among CM cases, as the lack of management of amphotericin-related toxicities remains a challenge in settings such as Lesotho. However, misdiagnosing early invasive cryptococcal disease as cryptococcal antigenaemia, or unmasking OIs, could also contribute to deaths in both arms (CrAg positive and negative). As it was not feasible to carry out a LP on all those with a CD4 <100 to identify early invasive disease, we sought to establish a safety net by closely following up all patients in the community, so that appropriate management could be instituted should symptoms develop.

The two deaths in confirmed CM cases occurred during the amphotericin course, and could be related to its toxicities, although this was not confirmed.

The main objective of the short-course (5–7 days of amphotericin B deoxycholate administration) strategy recommended by WHO is to reduce the risk of complications related to amphotericin B administration in places where laboratory monitoring is scarce or absent, like rural Lesotho [5]. There remains an urgent need for less toxic and more easily administrated treatments for CM, particularly in resource-poor settings where the proper monitoring of amphotericin B administration is challenging. Lipid formulations of amphotericin are less toxic, and are thus recommended for patients that are living with, or who are predisposed to, renal dysfunction [25]. However, the cost of treatment of this medication is too high for most resource-limited settings [26].

One death was observed among the asymptomatic CrAg-positive group. This patient may have already had *Cryptococcus* in the meninges, and developed meningitis after commencing fluconazole as an outpatient, although the patient also had TB and it was not possible to ascertain the actual cause of death. Since CrAg-positivity is associated with increased risk of death from TB [27], both diseases may have contributed, underscoring the need for concomitant screening for cryptococcal disease and TB in this population. Furthermore, interventions such as CrAg screening, which is designed to address mortality among late presenters, should also seek to improve the detection and management of TB.

A secondary consequence of this intervention was that additional cases of CM that would have previously gone undetected were now identified, exposing deficiencies in the clinical management of CM.

Our short follow up period (median 5 months) limited monitoring for CM development in asymptomatic CrAg-positive patients, despite pre-emptive treatment. However, our observation period covered the highest mortality risk period reported in similar resource-limited settings [28]. Additionally, we were unable to follow up those who were transferred out, and these patients may also have suffered adverse outcomes.

The lack of external quality control for an ideal comparison between lay counsellor’s performance and gold standard test (culture for *Cryptococcus sp*. in blood or CSF) was a limitation. Although, through the use of the CrAg-LFA positive quality control, we were able to ensure proper performance of each test batch.

Finally, the number of patients screened was small, so our estimated prevalence of CrAg positivity among individuals presenting with a CD4 count <100 cells/mm^3^ is subject to uncertainty. Nevertheless, only a few relatively small studies for POC CrAg screening have been carried out in Africa.

The public health importance of screening and early treatment is highlighted by the high frequency of CrAg positivity, as well as poor outcomes from treatment once fulminant CM develops. POC CrAg-LFA screening by a lay cadre and followed by pre-emptive fluconazole treatment for asymptomatic cases by nurses working in remote rural clinics of Lesotho (or referral to the hospital for symptomatic cases), proved feasible as a modest addition to overall clinic workload. However, close follow-up was needed to ensure proper management of cryptococcal disease and other OIs.

Further operational research in this area should address improved strategies for quality control, as well as patient referral and follow up. Given that TB is the principal cause of death among patients with advanced HIV disease, future work could also address how CrAg screening can be better integrated within a more comprehensive approach for the screening and management of people with advanced HIV disease.

The results of effective implementation of CrAg-LFA screening and monitoring of disease-free follow-up among those who received pre-emptive treatment suggest a positive impact of this intervention, particularly in a setting where the disease is largely undiagnosed and untreated. These early results support the wider use of CrAg-LFA POC screening in remote primary care settings.

In summary, this is as far as we are aware the only study that demonstrates the efficiency of lay counsellors performing CrAg-LFA screening, and documenting patient clinical outcomes. This is relevant within health systems where nurses have little capacity for additional activities, and where financial constraints limit the likelihood of more highly paid staff being employed to carry out this activity.
